# Supplementary material for: Improved Left Atrial Function in CRT Responders: A Systematic Review and Meta-Analysis
Source: J Clin Med. 2020 Jan 21;9(2):298. doi: 10.3390/jcm9020298 (PMC7074461; doi:10.3390/jcm9020298)
Supplement: Supplementary file 1 [file jcm-09-00298-s001.pdf]

Supplementary file

Improved Left Atrial Function in CRT Responders: A Systematic Review and Meta-Analysis

Ibadete Bytyçi [1,2], Gani Bajraktari [1,2], Per Lindqvist [1,3] and Michael Y. Henein [1,4,*]

[1] Institute of Public Health and Clinical Medicine, Umeå University, 90187 Umeå, Sweden; i.bytyci@hotmail.com (I.B.); gani.bajraktari@umu.se (G.B.); per.lindqvist@umu.se (P.L.)
[2] Clinic of Cardiology, University Clinical Centre of Kosovo, 10000, Prishtina, Kosovo
[3] Faculty of Medicine, Department of Surgical and Perioperative Sciences, Clinical Physiology, Umeå University, 90187 Umeå, Sweden
[4] Molecular and Clinic Research Institute, St George University, and Brunel University, SW17 0QT London, UK
* Correspondence: michael.henein@umu.se; Tel.: +46-90-785-14-31

Supplementary data

Table S1: Supplement 1. Summary of quality assessment analysis (Quality Assessment of Diagnostic Accuracy Studies-QUADAS 2).

Domain	Questions	Judgments
**Risk of bias** 1) Patient selection	*Was a consecutive or random sample of patients enrolled?*	Yes, No, Unclear
	Was a case-control design avoided?	Yes, No, Unclear
	Did the study avoid inappropriate exclusions?	Yes, No, Unclear
	Could the selection of patients have introduced bias?	Low, High, Unclear
**Applicability** 1) Patient selection	*Is there concern that the included patients do not match the review questions?*	Low, High, Unclear
**Risk of bias** 2) Index test	*Were the index test results interpreted without knowledge of the results of the reference standard?*	Yes, No, Unclear
	If a threshold was used, was it pre-specified?	Yes, No, Unclear
	Could the conduct or interpretation of the index test have introduced bias?	Low, High, Unclear
**Applicability** 2) Index test	*Is there concern that the index test, its conduct, or interpretation differ from the review question?*	Low, High, Unclear
**Risk of bias** 3) Reference standard	*Is the reference standard likely to correctly classify the target condition?*	Yes, No, Unclear
	Were the reference standard results interpreted?	Yes, No, Unclear
	without knowledge of the results of the index test?	Yes, No, Unclear
	Could the reference standard, its conduct, or its interpretation have introduced bias?	Low, High, Unclear
**Applicability** 3) Reference standard	*Is there concern that the target condition as defined by the reference standard does not match the review question?*	Low, High, Unclear
**Risk of bias** 4) Flow and timing	*Was there an appropriate interval between index test(s) and reference standard?*	Yes, No, Unclear
	Did all patients receive a reference standard?	Yes, No, Unclear
	Did patients receive the same reference standard?	Yes, No, Unclear
	Were all patients included in the analysis?	Yes, No, Unclear
	Could the patient flow have introduced bias?	Low, High, Unclear

Patients: Patients undergo CRT; Criteria for CRT.
Index test: Relationship between CRT responders and LA function
Comparator test (if applicable): Correlations of LA function parameters
Target condition: Correlations of LA function on related mean change of LVESV and/or LVEF;
Reference standard: Guidelines of heart failure and indications of Cardiac resynchronization therapy in heart failure.

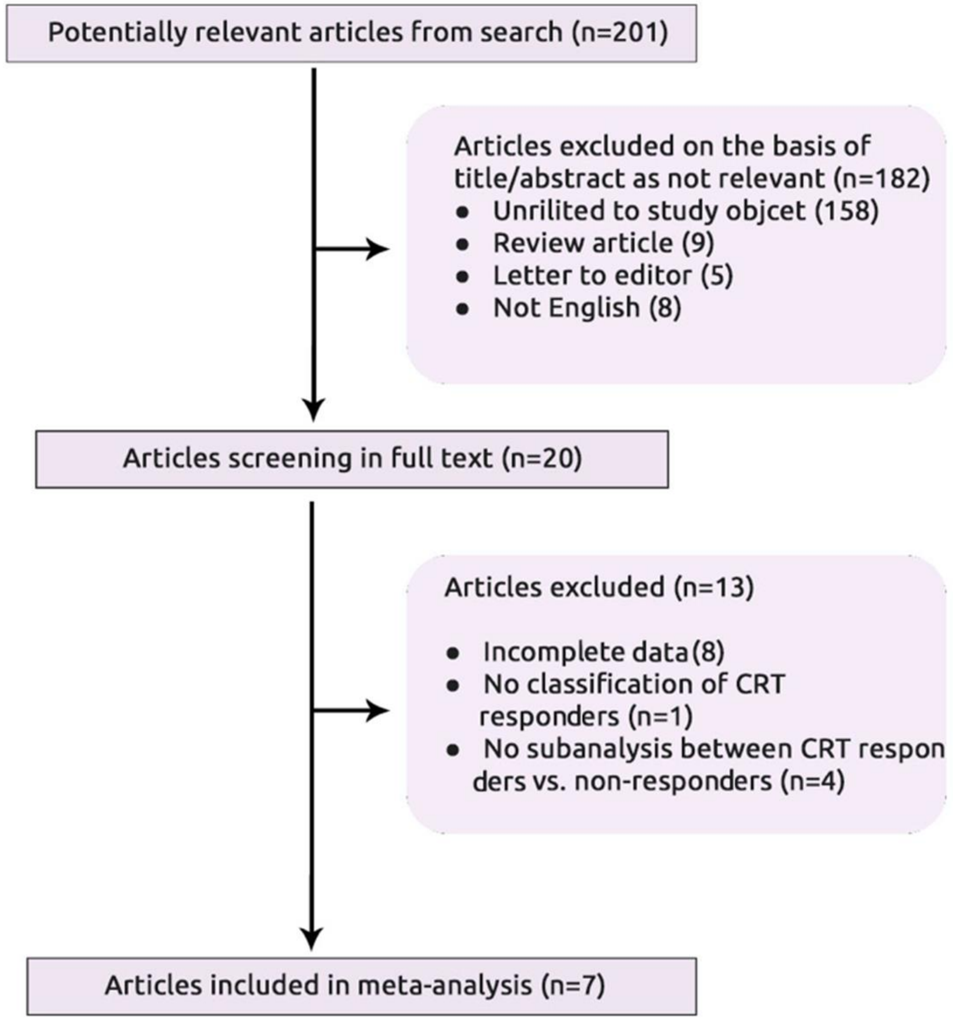

Figure 1. Supplement 2. Flow chart of study section**.**

Table S2: Supplement 3. Echocardiographic characteristics of LA functional measurements.

Study (Year)	Software	Platform	Probe (MHz)	FR	Gating	Observer	Tissue Tracking	LA function measurements	
								PALS	LA total EF
Yo 2007	GE Vivid 7	EchoPac	NR	NR	R-R	Two blinded	End-myocardial	Peak LA wall strain during LV systole	LAVmax-LAVmin /LAVmax
Marsan 2008	iE33 Philips	QLAB	NR	20–35	-	Single blinded	-	-	LAVmax-LAVmin /LAVmax
Donal 2009	GE Vivid 7	EchoPac	NR	NR	R-R	Two blinded	Endo-epicardial	Peak LA wall strain during LV systole	LAVmax-LAVmin /LAVmax
Fenon 2015	GE Vivid 9	EchoPac	2.5	60–80	R-R	Two blinded	Endocardial	Peak LA wall strain during LV systole	- -
Valenzia 2016	GE Vivid 7	EchoPac	3.5	NR	R-R	Single blinded	Endocardial	Peak LA wall strain during LV systole	- -
Badran 2017	GE Healthcare	EchoPac	2.5	NR	R-R	Single blinded	Endocardial	Peak LA wall strain during LV systole	LAVmax-LAVmin /LAVmax
Hansen 2017	GE Vivid 9	EchoPac	3–5	NR	-	Single blinded	-	-	LAVmax-LAVmin /LAVmax

a) Baseline of LVEDV in CRT responders vs. CRT non respondrers

Study or Subgroup	CRT responders Mean	SD	Total	CRT non-responders Mean	SD	Total	Weight	Mean Difference IV, Fixed, 95% CI	Year
Yu 2007	169	71	62	174	77	45	58.3%	-5.00 [-33.61, 23.61]	2007
Valzania 2016	177	60	18	178	69	12	20.8%	-1.00 [-48.88, 46.88]	2016
Badran 2017	255.5	77	24	251	67	13	21.0%	4.50 [-43.20, 52.20]	2017
**Total (95% CI)**			**104**			**70**	**100.0%**	**-2.18 [-24.01, 19.66]**	

Heterogeneity: Chi² = 0.11, df = 2 (P = 0.94); I² = 0%
Test for overall effect: Z = 0.20 (P = 0.85)

Mean Difference IV, Fixed, 95% CI

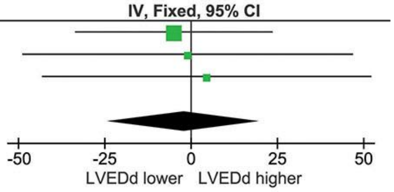

a) Baseline of LVESV in CRT responders vs. CRT non respondrers

Study or Subgroup	CRT responders Mean	SD	Total	CRT non-responders Mean	SD	Total	Weight	Mean Difference IV, Fixed, 95% CI	Year
Yu 2007	134	56	55	141	62	45	58.4%	-7.00 [-30.39, 16.39]	2007
Valzania 2016	129	47	18	128	53	12	23.3%	1.00 [-36.02, 38.02]	2016
Badran 2017	192	67	24	194	59	13	18.3%	-2.00 [-43.80, 39.80]	2017
**Total (95% CI)**			**97**			**70**	**100.0%**	**-4.22 [-22.10, 13.66]**	

Heterogeneity: Chi² = 0.14, df = 2 (P = 0.93); I² = 0%
Test for overall effect: Z = 0.46 (P = 0.64)

Mean Difference IV, Fixed, 95% CI

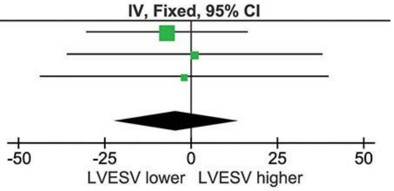

c) Baseline of LVEDd in CRT responders vs. CRT non respondrers

Study or Subgroup	CRT responders Mean	SD	Total	CRT non-responders Mean	SD	Total	Weight	Mean Difference IV, Fixed, 95% CI
Donal 2009	68.7	8.5	23	70	8.7	23	36.8%	-1.30 [-6.27, 3.67]
Feneon 2015	67	8	54	69	8	25	63.2%	-2.00 [-5.79, 1.79]
**Total (95% CI)**			**77**			**48**	**100.0%**	**-1.74 [-4.76, 1.27]**

Heterogeneity: Chi² = 0.05, df = 1 (P = 0.83); I² = 0%
Test for overall effect: Z = 1.13 (P = 0.26)

Mean Difference IV, Fixed, 95% CI

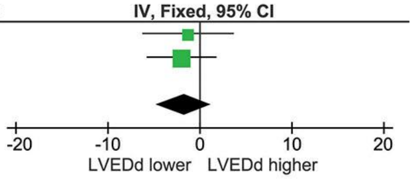

d) Baseline of LVEF in CRT responders vs. CRT non respondrers

Study or Subgroup	CRT responders Mean	SD	Total	CRT non-responders Mean	SD	Total	Weight	Mean Difference IV, Random, 95% CI
Badran 2017	25	7.4	24	24	6.4	13	27.9%	1.00 [-3.57, 5.57]
Donal 2009	26.6	6.7	23	22.4	6.6	23	31.0%	4.20 [0.36, 8.04]
Feneon 2015	28	3.1	54	30	2.4	25	41.1%	-2.00 [-3.25, -0.75]
**Total (95% CI)**			**101**			**61**	**100.0%**	**0.76 [-3.34, 4.86]**

Heterogeneity: Tau² = 10.23; Chi² = 10.03, df = 2 (P = 0.007); I² = 80%
Test for overall effect: Z = 0.36 (P = 0.72)

Mean Difference IV, Random, 95% CI

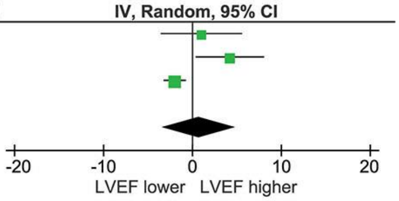

Figure S2: Supplement 4. Comparison of baseline LVEDV, LVESV, LVEDd and EF in group of patients with CRT responds vs. CRT non responders.

a) Baseline of QRS duration in CRT responders vs. CRT non responders

	CRT responders			CRT non-responders				Mean Difference
Study or Subgroup	Mean	SD	Total	Mean	SD	Total	Weight	IV, Fixed, 95% CI
Donal 2009	164	27	23	165	21	23	36.0%	-1.00 [-14.98, 12.98]
Feneon 2015	158	30	54	163	27	25	39.9%	-5.00 [-18.27, 8.27]
Valzania 2016	159	24	18	160	23	12	24.1%	-1.00 [-18.10, 16.10]
**Total (95% CI)**			**95**			**60**	**100.0%**	**-2.60 [-10.98, 5.79]**

Heterogeneity: Chi² = 0.21, df = 2 (P = 0.90); I² = 0%
Test for overall effect: Z = 0.61 (P = 0.54)

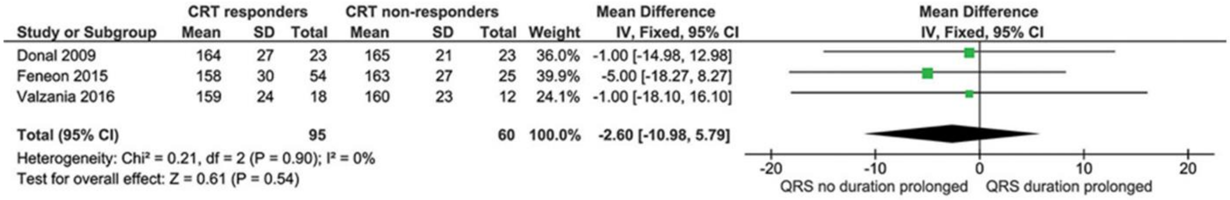

a) Baseline of LAD in CRT responders vs. CRT non responders

	CRT responders			CRT non-responders				Mean Difference	
Study or Subgroup	Mean	SD	Total	Mean	SD	Total	Weight	IV, Random, 95% CI	Year
Yu 2007	52.2	8.8	62	55	9.9	45	34.1%	-2.80 [-6.43, 0.83]	2007
Donal 2009	53	8	23	50	8	23	30.4%	3.00 [-1.62, 7.62]	2009
Badran 2017	44	1.8	24	50	5.8	13	35.5%	-6.00 [-9.23, -2.77]	2017
**Total (95% CI)**			**109**			**81**	**100.0%**	**-2.17 [-7.01, 2.67]**	

Heterogeneity: Tau² = 14.47; Chi² = 9.78, df = 2 (P = 0.008); I² = 80%
Test for overall effect: Z = 0.88 (P = 0.38)

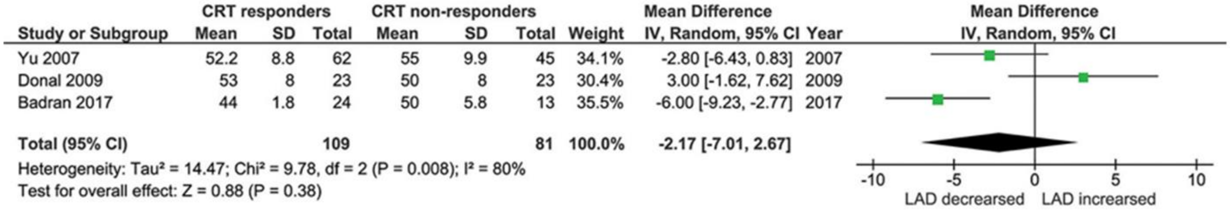

Figure S3: Supplement 5. Comparison of baseline QRS duration and LA dimension in group of patients with CRT responders vs. CRT non responders.

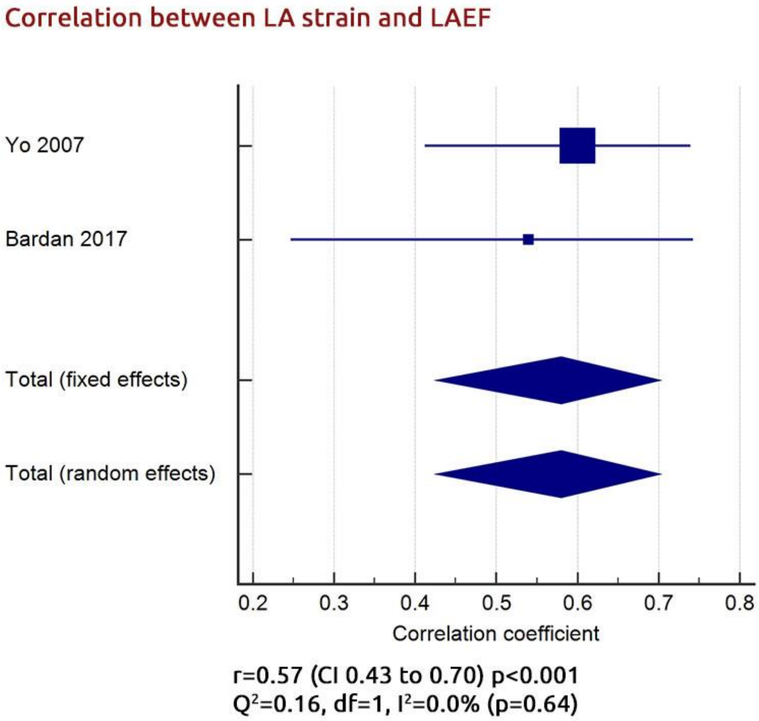

Figure S4: Supplement 6. Weighted summary correlation between LA strain and LAEF.

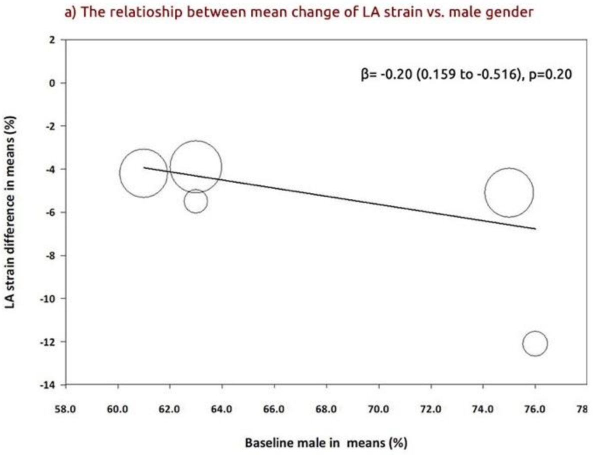

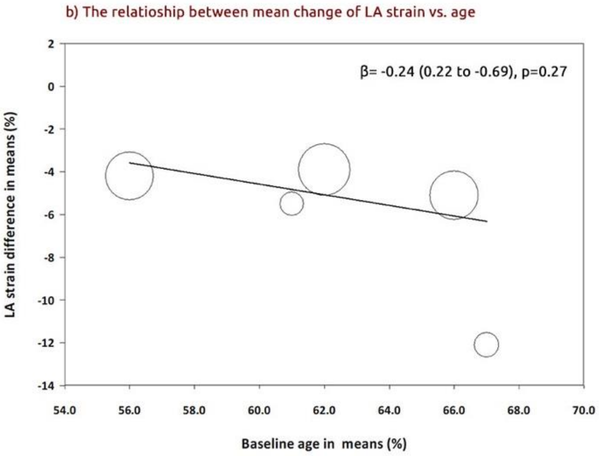

Figure S5: Supplement 7. Relationship between LA strain change and a) male gender; b) age.

Table S3: Supplement 8. Summary of QUADAS-2 Assessment of Selected Studies.

**Author (year) reference**	**Risk of bias**				**Applicability concerns**		
	**Patients** selection	**Index** test	**Reference** standard	**Flow and** timing	Patients selection	Index test	Reference standard
Yo 2007	Low	Low	Low	Low	Low	Low	Low
Marsan 2008	Low	Low	Low	Low	Low	Low	Low
Donal 2009	Low	Unclear	Low	Low	Low	Unclear	Unclear
Feneon 2015	Low	Unclear	Unclear	Unclear	Unclear	Unclear	Unclear
Valenzia 2016	Low	Low	Low	Low	Low	Low	Low
Badran 2017	Low	Low	Low	Low	Low	Low	Low
Hansen 2017	Unclear	Low	High	High	Unclear	Low	High

QUADAS-2: Quality Assessment of Diagnostic Accuracy Studies-2.

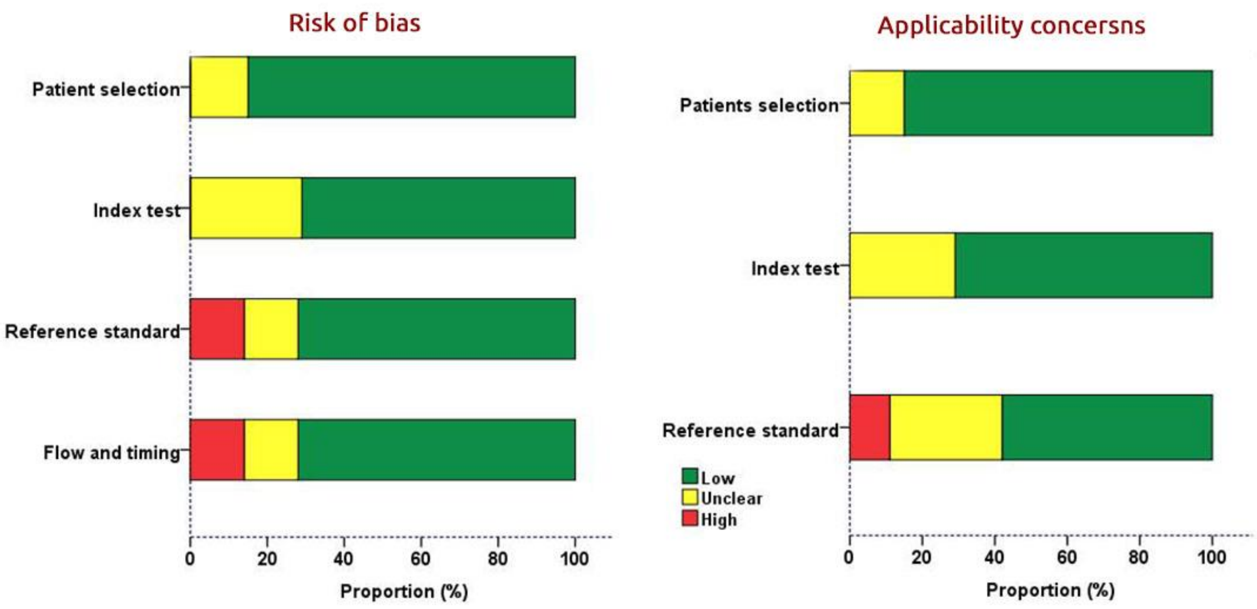

Figure 6. Supplement 9. Summary of QUADAS-2 Assessment of Selected Studies.
